# Case report: retroperitoneal aspergilloma in a patient with rheumatoid arthritis presenting as malignant tumor

**DOI:** 10.1186/s12894-018-0381-0

**Published:** 2018-07-31

**Authors:** Risa Shinoki, Teppei Takeshima, Koichiro Horie, Toshihiro Matsui, Ayako Horita, Ikuo Saito, Kotaro Hirai

**Affiliations:** 1 Department of Urology, National Hospital Organization Sagamihara National Hospital, Sagamihara, Kanagawa Japan; 2 Department of Rheumatology, National Hospital Organization Sagamihara National Hospital, Sagamihara, Kanagawa Japan; 3 Department of Pathology, National Hospital Organization Sagamihara National Hospital, Sagamihara, Kanagawa Japan; 4 Department of Urology, National Hospital Organization Yokohama Medical Center, Yokohama, Kanagawa Japan

**Keywords:** Aspergilloma, Immunocompromised host, Rheumatoid arthritis with vasculitis, Retroperitoneum, Surgical resection

## Abstract

**Background:**

Aspergillosis in patients with impaired immunity usually presents with invasive pulmonary infection and dissemination to a variety of organs via hematogenous spread. Aspergilloma in the retroperitoneal cavity is a rare disease with only a few cases reported in the literature. To the best of our knowledge, the present case of a retroperitoneal aspergilloma with no surgical history is only the second report in the literature.

**Case presentation:**

A 65 year-old man, who had been receiving immunosuppressive treatment for rheumatoid arthritis with vasculitis for 9 years, was referred to the Urology Department with a retroperitoneal mass. This was confirmed by computed tomography performed during treatment for pulmonary aspergilloma. Because it was not possible to rule out malignant disease (e.g., liposarcoma), surgical exploration was performed. Pathological examination revealed aspergillus hyphae with fat necrosis, and retroperitoneal aspergilloma was diagnosed and appropriately treated. The tumor did not recur subsequently.

**Conclusion:**

Our present case emphasizes that pharmacological treatments for aspergilloma in the retroperitoneal cavity have poor drug transitivity, so the relative effectiveness of pharmacological response is not useful for differentiating retroperitoneal aspergilloma from malignant disease.

## Background

Aspergillosis, alongside candidiasis, is one of the most common deep-seated mycoses. The increased number of immunocompromised patient resulting from the use of immunosuppressive drugs for organ transplantation and other indications, as well as the effects of anticancer chemotherapy, has resulted in an increased incidence of opportunistic aspergillosis [1, 2]. Aspergillomas are most commonly found in the lungs, where *Aspergillus* colonizes a cyst or existing cavernous lesion and multiplies, forming a fungus ball. Long-term ingestion of antifungal agents is necessary to treat pulmonary aspergilloma and surgical resection may be indicated for isolated lesions when they are refractory to antifungal treatment.

Here, we report a rare case of retroperitoneal aspergilloma successfully treated by surgical resection after pharmacological therapy.

## Case presentation

A retroperitoneal mass on the left side was found in a 65-year-old-man who was then referred to the Urology Department. He had been receiving methylprednisolone and cyclosporine as treatment for rheumatoid arthritis with vasculitis in the Rheumatology Department of the same hospital. He complained of exertional dyspnea and was hospitalized on suspicion of atypical pneumonia. Blood tested positive for both β-D-glucan and aspergillus antigen. A diffuse nodular shadow across both lungs was seen on chest computed tomography (CT) and a diagnosis of pulmonary aspergilloma was made. Treatment with voriconazole (200 mg twice a daily) was initiated.

On subsequent CT 8 months thereafter, the nodular shadow in the lungs appeared smaller, but a mass in the left retroperitoneum was now seen. Consequently, he was referred to the Urology Department. His past medical history included rheumatoid arthritis with vasculitis, steroid-induced diabetes, hyperlipidemia, and compression fracture of the lumbar vertebrae. He had been receiving methylprednisolone and cyclosporine treatment for 8 years. In addition, he received bezafibrate and his diabetes was controlled with hypodermic insulin and oral sitagliptin. He had no notable family history. The patient’s physical findings were normal, except for chest and joint symptoms related to rheumatoid arthritis with vasculitis.

**Fig. 1:**
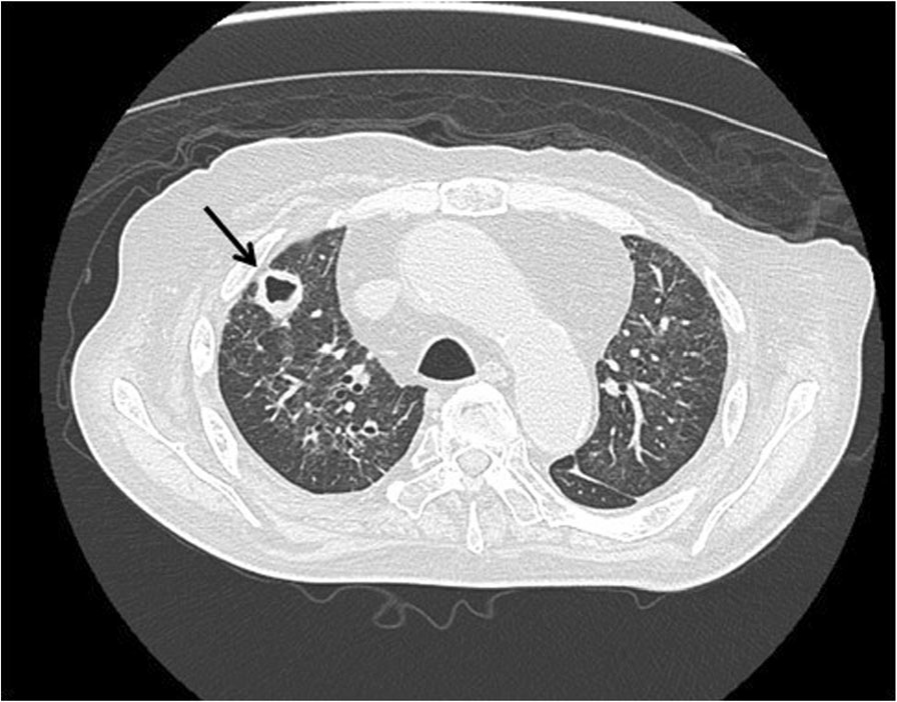
Hollow nodules seen on chest CT

**Fig. 2:**
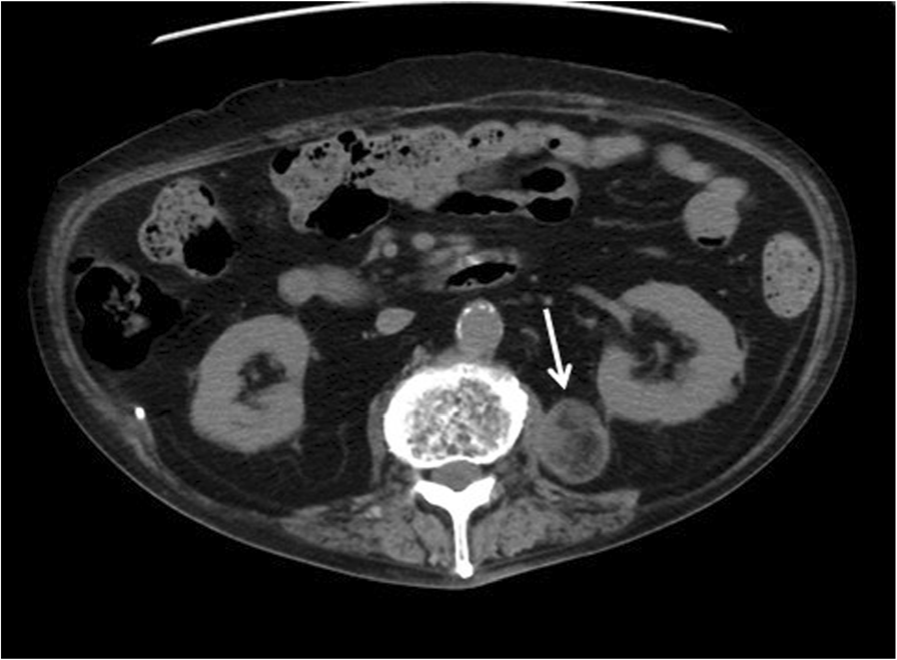
Abdominal CT image showing a 30 mm × 35 mm mass in the left retroperitoneal cavity

Blood test results on admission were as follows: white blood cells, 11830/μL; C-reactive protein, 2.16 mg/dL; glucose, 250 mg/dL; total-cholesterol, 230 mg/dL; triglyceride, 642 mg/dL. There was no apparent liver or kidney dysfunction. The aspergillus antigen level was 0.7 (positive, > 0.5) and the β-D glucan level was 240 pg/ml (> 11.0). The frequent occurrence of a diffuse nodular shadow across both lungs on a simple CT image of the chest was noted. Thus, intravenous voriconazole was commenced. On subsequent CT 3 weeks later, a reduction in the pulmonary nodular shadow and improvement of the previous pneumonia was identified (Fig. 1). The voriconazole dose was altered for oral application (200 mg/day). He was then referred to the Urology Department once more because a 35-mm mass was found in the retroperitoneal cavity on CT (Fig. 2).

**Fig. 3:**
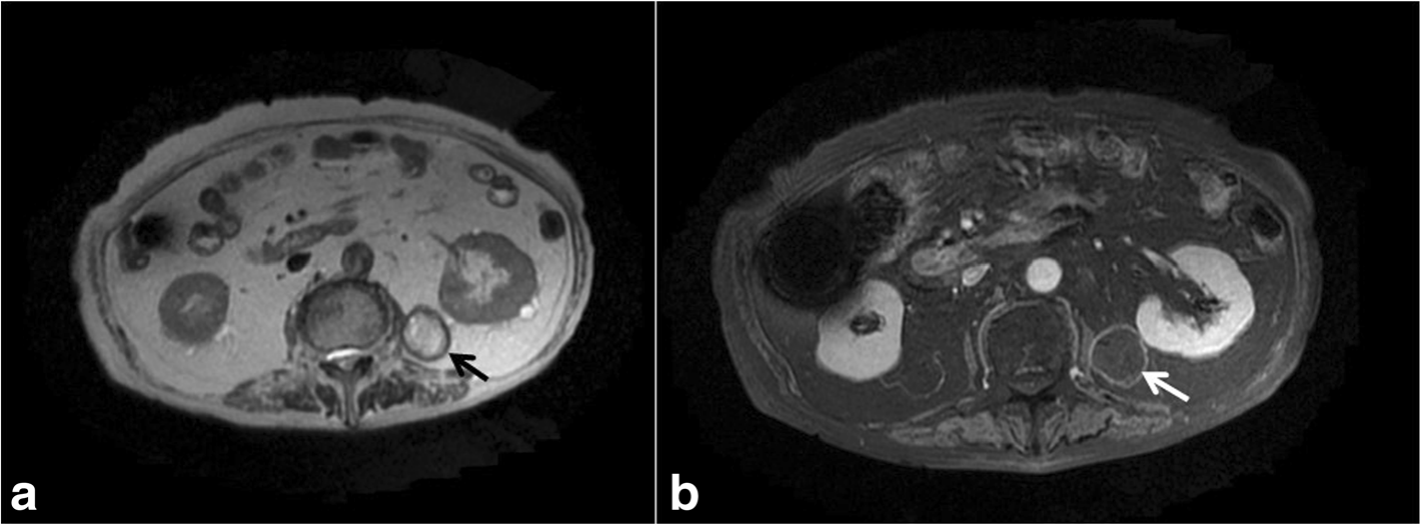
MRI of the abdomen and pelvis: **a** axial T2. **b** Fat suppression axial T1 images. The mixed heterogeneous signal of the high-low signal in both the T2 and T1-weighted images, and signal strength, decreased with T1 fat suppression

The tumor did not exhibit any internal contrast uptake and a film-like layer of contrast was seen around the border on abdominal contrast-enhanced CT. As a result, we believed that the tumor was not a renal angiomyolipoma; however, it was considered possible that it was a lipoma, liposarcoma, or teratoma. We recognized the mixed heterogeneous nature of the high-low signal on abdominal magnetic resonance imaging in both the T2 and T1-weighted images, with signal strength decreasing on the T1 fat suppression images. As liposarcoma could not be ruled out, a left retroperitoneal tumor resection was performed (Fig. 3).

We performed a lumbar incision under the left 12th rib with the patient in the jackknife posture during surgery. The mass did not adhere to the surrounding tissues, so there was a small amount of blood loss.

**Fig. 4:**
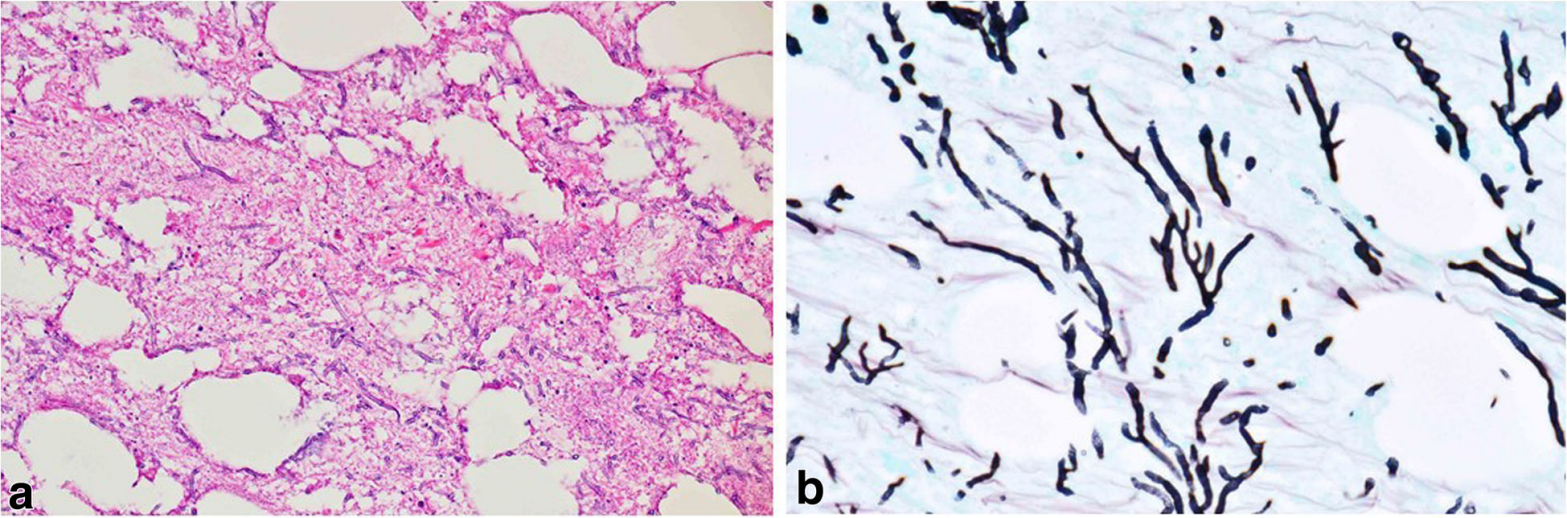
Microscopic findings (hematoxylin and eosin stain). **a** Tumor identified as adipose tissue and necrotic debris encapsulated by fibrous connective tissue. **b** Large number of fungi in the mass

Histological examination identified the contents of tumor as adipose tissue and necrotic debris that were encapsulated by fibrous connective tissue, with a large number of fungal hyphae in the tumor. A blood vessel cavity that passed through the necrotic tissue was identified as the origin (Fig. 4). Because the origin showed a Y-shaped divergence with the form of the constituent part, the histopathological diagnosis was aspergilloma. The postoperative course was uneventful and the patient was discharged 10 days after surgery. Thereafter, he continued antifungal therapy and no recurrence was seen on follow-up CT. In the tenth month after the operation, he developed pancytopenia, thought to be related to bone marrow suppression, with Cytomegalovirus infection. He suffered respiratory failure and succumbed.

## Discussion and conclusion

Aspergilloma is one of the clinical manifestations of aspergillosis, with pulmonary aspergilloma being the main presentation. In an aspergilloma, *Aspergillus* forms a ball of fungus in an existing lung cavity. Aspergilloma formation in an abdominal organ is rare. Several examples have been reported elsewhere, including the paranasal sinus and central nervous system, as well as formation of a fungus ball in the abdominal cavity after abdominal surgery. To the best of our knowledge, our case of an aspergilloma that formed in the retroperitoneal cavity in a patient with no surgical history is the second case globally [3–5]. Mycobacterial infectious disease often occurs in the abdominal cavity of immune-deficient patients, such as diabetics and long-term steroid users, both the case for the patient described here [5, 6]. Because pharmacological treatments for aspergilloma, such as micafungin or voriconazole, have poor drug transitivity in cavities, surgical resection is an option in cases refractory to antifungals. The failure of antifungal treatment could have been due to a difference of the blood concentration at each lesion.

The reason why aspergillus organisms located in the retroperitoneal space survive longer remains unclear. Aspergilloma mainly affects people with underlying cavitary lung diseases such as tuberculosis, sarcoidosis, bronchiectasis, cystic fibrosis and systemic immunodeficiency. The fungus colonizes a cavity and is able to grow free of interference because critical elements of the immune system as well as anti-fungal agents are unable to penetrate into the cavity. Following colonization, angioinvasion and dissemination could be occurring in immunocompromised patients. These considerations suggest the possibility of prior retroperitoneal infection such as with bacillus tuberculosis, which more commonly form abscesses in the retroperitoneal cavity.

The reason that aspergilloma mimicked liposarcoma may be that the tumor contained heterogeneous mixtures of adipose tissue and necrotic debris, appearing similar to some liposarcomas (e.g. myxoid liposarcoma).

In this case, even though the patient had already received voriconazole treatment previously which had reduced the pulmonary lesion, the retroperitoneal lesion appeared to be increasing in size. The disease course did not allow us to rule out a retroperitoneal malignant tumor for which the treatment of choice is surgical resection, as same as previous reports. This led to the appropriate treatment for this patient despite an erroneous indication. We have presented a very rare case of retroperitoneal aspergilloma, thought to be only the second in the literature. Thus, physicians should bear in mind the possibility of failure of anti-mycotic treatment when diagnosing such immuno-deficient patients, and should select appropriate treatment.

## Abbreviation

CT: Computed tomography
